# Identification and validation of potential diagnostic signature and immune cell infiltration for HIRI based on cuproptosis-related genes through bioinformatics analysis and machine learning

**DOI:** 10.3389/fimmu.2024.1372441

**Published:** 2024-04-16

**Authors:** Fang Xiao, Guozhen Huang, Guandou Yuan, Shuangjiang Li, Yong Wang, Zhi Tan, Zhipeng Liu, Stephen Tomlinson, Songqing He, Guoqing Ouyang, Yonglian Zeng

**Affiliations:** ^1^ Division of Hepatobiliary Surgery, The First Affiliated Hospital of Guangxi Medical University, Nanning, China; ^2^ Key Laboratory of Early Prevention and Treatment for Regional High Frequency Tumor (Guangxi Medical University), Ministry of Education, Nanning, China; ^3^ Guangxi Key Laboratory of Immunology and Metabolism for Liver Diseases, Nanning, China; ^4^ Department of Microbiology and Immunology, Medical University of South Carolina, Charleston, SC, United States

**Keywords:** cuproptosis, hepatic ischemia and reperfusion injury, immune infiltration, machine learning, immune microenvironment

## Abstract

**Background and aims:**

Cuproptosis has emerged as a significant contributor in the progression of various diseases. This study aimed to assess the potential impact of cuproptosis-related genes (CRGs) on the development of hepatic ischemia and reperfusion injury (HIRI).

**Methods:**

The datasets related to HIRI were sourced from the Gene Expression Omnibus database. The comparative analysis of differential gene expression involving CRGs was performed between HIRI and normal liver samples. Correlation analysis, function enrichment analyses, and protein-protein interactions were employed to understand the interactions and roles of these genes. Machine learning techniques were used to identify hub genes. Additionally, differences in immune cell infiltration between HIRI patients and controls were analyzed. Quantitative real-time PCR and western blotting were used to verify the expression of the hub genes.

**Results:**

Seventy-five HIRI and 80 control samples from three databases were included in the bioinformatics analysis. Three hub CRGs (NLRP3, ATP7B and NFE2L2) were identified using three machine learning models. Diagnostic accuracy was assessed using a receiver operating characteristic (ROC) curve for the hub genes, which yielded an area under the ROC curve (AUC) of 0.832. Remarkably, in the validation datasets GSE15480 and GSE228782, the three hub genes had AUC reached 0.904. Additional analyses, including nomograms, decision curves, and calibration curves, supported their predictive power for diagnosis. Enrichment analyses indicated the involvement of these genes in multiple pathways associated with HIRI progression. Comparative assessments using CIBERSORT and gene set enrichment analysis suggested elevated expression of these hub genes in activated dendritic cells, neutrophils, activated CD4 memory T cells, and activated mast cells in HIRI samples versus controls. A ceRNA network underscored a complex regulatory interplay among genes. The genes mRNA and protein levels were also verified in HIRI-affected mouse liver tissues.

**Conclusion:**

Our findings have provided a comprehensive understanding of the association between cuproptosis and HIRI, establishing a promising diagnostic pattern and identifying latent therapeutic targets for HIRI treatment. Additionally, our study offers novel insights to delve deeper into the underlying mechanisms of HIRI.

## Introduction

Hepatic ischemia and reperfusion injury (HIRI) represents a significant complication observed in diverse clinical scenarios, including liver resection, liver transplantation, and trauma. In liver transplantation, HIRI plays a crucial role as a risk factor for acute and chronic transplant rejections, as well as primary graft dysfunction or non-function. These detrimental consequences can be particularly prominent in liver transplants involving broader criteria, which encompass organs from marginal, deceased, and non-heart-beating donors (1). HIRI involves a biphasic pathophysiological progression: the first phase involves ischemia-induced injury, which is marked by the depletion of ATP and glycogen, along with cellular metabolic stress resulting from mitochondrial dysfunction, ultimately resulting in initial cell death. Subsequently, reperfusion injury occurs upon restoration of blood flow and reoxygenation. Throughout this process, metabolic irregularities, coupled with an abundance of reactive oxygen species (ROS) and cytokines or chemokines, activate various immune cells, triggering a deep-seated inflammatory response that results in severe liver damage (2, 3).

Copper, an essential micronutrient, is a crucial catalytic cofactor in a wide spectrum of biological processes, including mitochondrial respiration, the synthesis of biocompounds, and antioxidant defense (4). Maintaining systemic copper levels within a narrow range is essential to support normal biochemical functions, as even slight elevations can trigger cytotoxicity or cell death. Imbalances in copper levels can contribute to the development of a range of diseases, such as Wilson’s disease, Menkes disease, cancer, and inflammatory diseases (5–8). The mechanisms and exact forms of cell death induced by copper have remained mysterious for a considerable period. However, copper-induced cell death has recently been officially recognized as a unique form of programmed cell death, termed “cuproptosis.” Cuproptosis operates independently of established cell death pathways, including autophagy, ferroptosis, apoptosis, and proptosis. Within cells, Copper directly binds to lipoylated components within the tricarboxylic acid (TCA) cycle, leading to the clustering of lipoylated proteins and the loss of iron-sulfur (Fe-S) clusters, thereby triggering proteotoxic stress and ultimately resulting in cell death (9)..

The liver plays a central role in regulating copper metabolism, overseeing the processes of copper absorption, storage, distribution, transport, and excretion. Copper and cuproptosis are closely associated with liver disease (10). Wilson’s disease exemplifies the excessive accumulation of copper in the liver, attributable to a mutation in ATP7B. This mutation has been reported to potentially involve cuproptosis (8). Some studies have also hinted at an underlying connection between cuproptosis and other liver diseases such as hepatocellular carcinoma and nonalcoholic fatty liver disease; cuproptosis has also attracted great interest in its therapy (11, 12). However, research on the role of copper or cuproptosis in HIRI remains relatively limited. In a rat model of HIRI, it was noted that marginal copper deficiency could lead to an increase in hepatic neutrophil accumulation, indicating a potential role for copper in modulating the inflammatory response to HIRI (13). Nevertheless, because the precise role of cuproptosis in HIRI is still unclear, further investigation is warranted to explore the potential of cuproptosis as a therapeutic target for HIRI. In this study, we analyzed differentially expressed cuproptosis-related genes (CRGs) and their immune characteristics between 75 HIRI patients and 80 control cases. Machine learning algorithms were used to identify key genes that could aid in diagnostic predictions. The predictive model was validated through the utilization of a nomogram, calibration curve, receiver operating characteristic (ROC) curve, and decision curve analysis (DCA). Furthermore, we investigated the correlation between hub CRGs and immune cell infiltration. Lastly, we established potential target drugs and constructed competitive endogenous RNA (ceRNA) networks in this investigation.

## Materials and methods

### Patients and datasets

**Figure 1:**
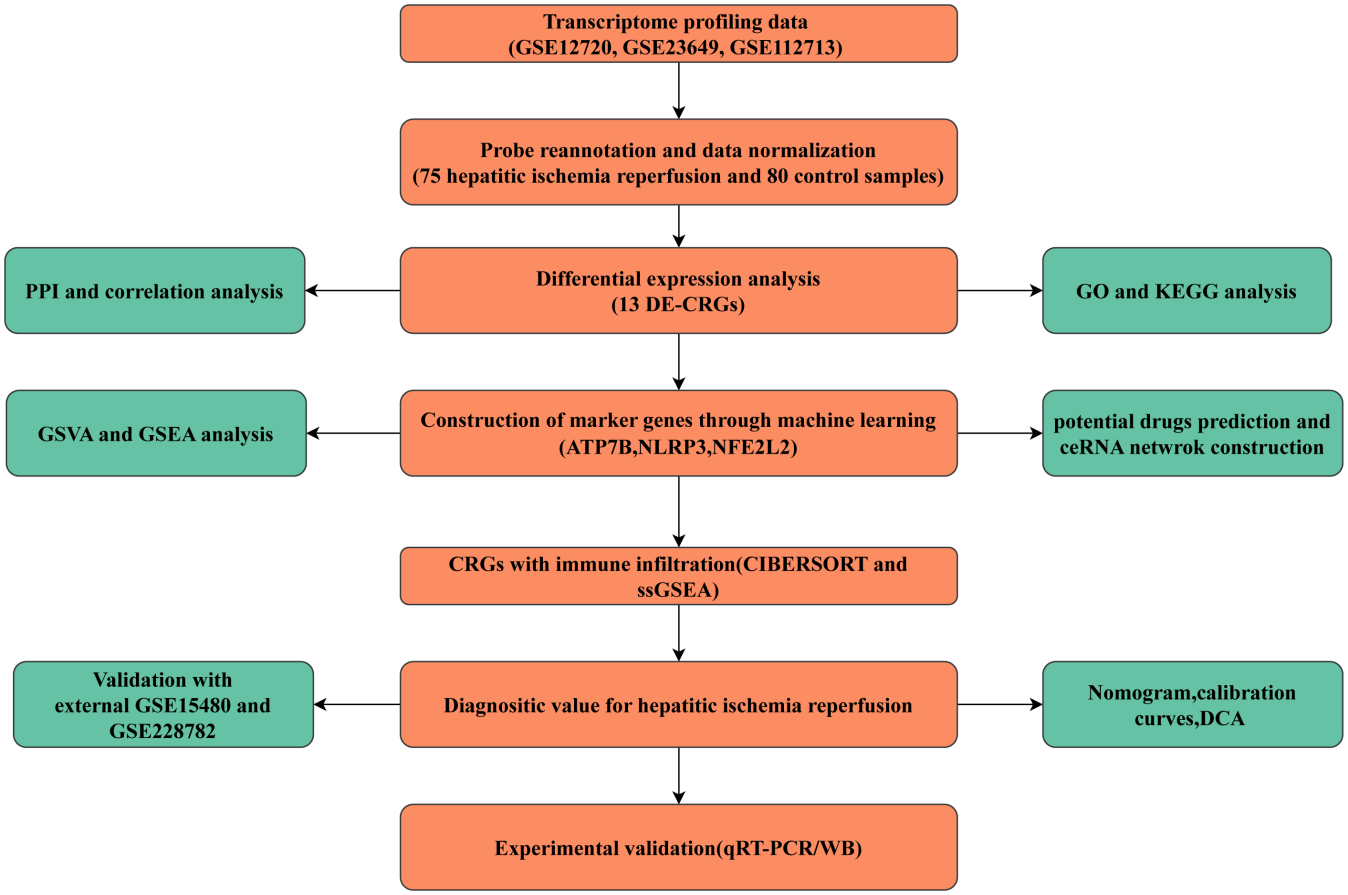
Flowchart of the present study.

Transcriptome profiling data for samples with hepatic ischemia-reperfusion injury (HIRI) and control samples (non-HIRI) were obtained from three datasets available in the Gene Expression Omnibus (GEO) database. namely GSE12720 (21 HIRI and, 21 control cases), GSE23649 (32 HIRI and, 33 control cases), and GSE112713 (22 HIRI and, 26 control cases), GSE15480 (6 HIRI and, 6 control cases), GSE228782 (9 HIRI and, 12 control cases). The combined datasets encompassed 75 cases of HIRI and 80 control cases. Thirty-eight CRGs were recovered from the previous literature (*ATP7A, ATP7B, ACO2, CDKN2A, DBT, DLAT, DLD, DLST, DPYD, FDX1, GCSH, GLRX5, GLS, ISCA2, LIAS, LIPA, LIPT1, LIPT2, LIPM, MTF1, NDUFA1, NDUFA8, NDUFB10, NDUFB2, NDUFB6, NLRP3, NDUFC1, NDUFC2, NDUFV2, NFE2L2, PDHA1, PLAT, PDHB, POLD1, PPAT, SLC31A1, SDHB, and TIMMDC1*) (11, 12). ATP7A mutations cause Menkes disease, Hence, ATP7A was excluded from further consideration. The flowchart of the present study is shown in **Figure 1**.

### Expression of differential genes related to CRGs in HIRI

Wilcoxon’s signed-rank test was adopted to identify DEGs in CRGs between the HIRI and control groups. A boxplot of DEGs from 37 CRGs was created using the R package “ggpubr.” The results were subsequently represented as volcano and heatmap plots with the R packages “ggplot2” and “pheatmap.” The intersection of DEGs linked to CRGs was generated using the R package “VennDiagram” and is referred to as DEG-CRGs for further analysis. *p* < 0.05 was considered to indicate significant differences.

### Correlation analysis and PPI network construction

The landscape of the 23 chromosomes and a heatmap of 13 DE-CRGs were created using the R packages “RCircos” and “heatmap,” respectively. A correlation Circos plot was generated using the “circlize” package, drawing upon Pearson correlation analysis between DEG-CRGs. The STRING database (https://string-db.org/) was utilized to construct a protein-protein interaction (PPI) network of 13 DE-CRGs. PPI analysis was conducted with a medium confidence level of 0.4.

### Gene Ontology and KEGG pathway enrichment analysis of DEG-CRGs

Gene Ontology (GO) analysis, encompassing Molecular Function (MF), Cellular Component terms, and Biological Process, was performed on 13 differentially expressed CRGs. Additionally, Kyoto Encyclopedia of Genes and Genomes (KEGG) pathway analyses were conducted. These analyses were executed using the R package “clusterProfiler” and the results were then visualized with the aid of the R package “enrichplot.”

### Construction of the CRG diagnostic model

Three datasets, GSE12720, GSE23649, and GSE112713, were merged together as training data, and the GSE15480 and GSE228782 datasets served as the validation data for the machine learning model. Identification of the hub genes for HIRI prognosis prediction was performed using support vector machine–recursive feature elimination (SVM–RFE) (SVM-RFE), random forest (RF) algorithm, and least absolute shrinkage and selection operator (LASSO) regression methods. Using the R package “glmnet,” LASSO regression was applied to the selected linear model to reduce data dimensions while retaining valuable variables. The optimal value for building the model was determined by setting the minimum lambda value. SVM-RFE, a supervised machine learning model, is capable of discerning between positive and negative instances by iteratively eliminating feature vectors generated through SVM. The SVM-RFE model was implemented using the R package “e1071” to identify the most influential genes. The SVM-RFE approach was employed to determine the optimal variables by looking for the iteration corresponding to the smallest cross-validation error. The RF model is an ensemble machine learning approach to determine the optimal number of variables by constructing multiple independent decision trees. An RF model with “ntree value” set to 500 was constructed using the R package “randomForest.” In the current study, the intersection method was used to identify the most robust hub genes, which were derived from SVM–RFE, RF, and LASSO algorithms.

### ROC and nomogram model construction

The diagnostic performance of the marker genes was assessed by calculating time-dependent ROC curves, which measured metrics such as the area under the curve (AUC), sensitivity, and specificity. ROC curve analysis was conducted using the R software package “pROC.” Furthermore, the diagnostic model’s ability to accurately diagnose was further validated by utilizing the external GSE15480 and GSE228782 datasets to evaluate the diagnostic potential of the hub genes. The datasets have undergone normalization procedures and batch effects have been removed between them.

We constructed a nomogram model using the R package “rms” to predict the risk of HIRI. Each hub gene had a distinct score, which was then added up to form a total score. The predictive power of the model was assessed using a calibration curve. Additionally, this model was evaluated for its clinical usefulness using clinical impact curves and decision curves.

### Gene set enrichment analysis and gene set variation analysis

To investigate the potential role of the central genes, we employed the Gene Set Enrichment Analysis (GSEA) functionality provided by the “clusterProfiler” R package. Among the available options, we chose the KEGG gene set (c2.cp.kegg.symbols.gmt) sourced from the Molecular Signatures Database as our reference. Statistical significance was configured at p < 0.05 for the enrichment analysis. In order to visually represent the distinctive enrichment patterns of gene sets between the high- and low-expression subtypes, we performed gene set variation analysis (GSVA) utilizing the “GSVA” R package. Furthermore, differential expression pathways were further elucidated by comparing GSVA scores between the low- and high-expression subtypes using the “limma” R package.

### Evaluating the immune infiltration

The CIBERSORT algorithm was used to estimate the fractions of 22 types of human immune cells in each sample from the merged dataset (14). An accurate immune cell fraction was considered significant with a p-value of less than 0.05. In particular, for each sample, the sum of fractions of the 22 immune cell types was normalized to a total value of 1. Single-sample gene set enrichment analysis (ssGSEA) was conducted using the R package “GSVA” to evaluate each sample’s enrichment score of infiltrating immune cells and immune-related functions. From the ImmPort database (http://www.immport.org), the reference gene set was obtained. Spearman’s correlation analysis was used to determine the correlation between the center gene and the immune score, and the Wilcoxon test was used to estimate the difference in immune cell enrichment scores and immune-related functions between the two groups. A boxplot was used to visualize the composition of the enrichment score between the HIRI and control groups.

### Identification of potential small molecule drugs

Drug-gene interaction databases (DGIdb, https://dgidb.genome.wustl.edu/) encompass online repositories that furnish comprehensive information on interactions between drugs and genes/proteins, meticulously curated from diverse sources such as The Druggable Genome and Therapeutic Targets Database (15). Additionally, the Drug Signatures Database (DSigDB) was employed to prognosticate putative pharmaceutical agents associated with the three central genes, gaining access to DSigDB via the Enrichr website (https://amp.pharm.mssm.edu/Enrichr/). Both DGIdb and DSigDB were leveraged to anticipate potential drugs that possess the capability to selectively interact with the designated marker genes. The visually compelling drug-gene network derived from DGIdb was elegantly depicted utilizing the Cytoscape software.

### ceRNA network construction

Based on hub genes, the miRDB (http://www.mirdb.org/) and TargetScan (http://www.targetscan.org/vert_80/) databases were used to anticipate miRNA–mRNA interactions. SpongeScan (http://spongescan.rc.ufl.edu/) combine evidence for a direct interaction between the predicted miRNA and lncRNA. A ceRNA mRNA–miRNA–lncRNA network was created and visualized using Cytoscape (version 3.9.0).

### HIRI mouse model establishment and histological procedure

Eight-week-old male C57BL/6J mice were subjected to total warm hepatic ischemia and reperfusion procedures: The hepatic artery and portal vein were clamped with a microaneurysm clamp to restrict blood flow for 30 minutes, inducing hepatic ischemia. After the clamp was removed, hepatic reperfusion was initiated. After 6 hours of reperfusion, mice were euthanized to obtain serum and liver samples. The serum levels of ALT and AST were measured using analytical kits from Sigma-Aldrich, following the manufacturer’s guidelines. Fresh liver tissue was immersed in a solution of 4% paraformaldehyde for embedding in paraffin. Sections of 4 μm thickness were obtained using a microtome and subsequently stained with H&E and MPO (abcam) according to the manufacturer’s protocol. All animal experiments were approved by the Animal Care and Use Committee of Guangxi Medical University according to the 1996 National Research Council guide for the care and use of laboratory animals. Mice were given a pelleted diet and ad libitum water and maintained on a 12-hour light/dark cycle.

### RNA extraction, quantitative real-time PCR

The RNA was isolated from HIRI and control mouse liver samples utilizing TRIzol reagent (Thermo Fisher Scientific, USA), followed by cDNA synthesis using the PrimeScript™ RT reagent kit (Takara, Japan). Quantitative real-time PCR (qRT-PCR) analysis was conducted employing the FX Connect system (Bio-Rad, USA) and SYBR^®^ Green Supermix (Bio-Rad, USA). The expression levels of the pivotal genes were evaluated through the 2−ΔΔCT method with GAPDH serving as the internal reference control. The primer sequences utilized in the qRT-PCR experiments are provided in **Supplementary Table 1**.

### Western blot analysis

Fresh liver tissue samples obtained from both HIRI and wild-type mice were homogenized on ice using a lysis buffer (Sigma-Aldrich) that contained a protease inhibitor cocktail (from MCE). The homogenates underwent sonication and were subsequently centrifuged at 10,000 g and 4°C to eliminate any cellular debris. Protein concentrations were then determined, and samples containing the same amounts of protein in equal volumes of sample buffer were separated using a 4–15% Tris-HCl polyacrylamide gel and transferred onto a PVDF membrane (Bio-Rad). To prevent nonspecific binding, Tris-buffered saline containing 5% non-fat dry milk was used to block nonspecific binding sites for 1 hour at room temperature. In addition, the PVDF membrane was also blocked with Tris-buffered saline and 5% non-fat milk for 1 hour at room temperature to further minimize nonspecific binding. Membranes were then incubated with antibodies to NLRP3, ATP7B, and NFE2L2 (Proteintech), or GAPDH (Santa Cruz Biotechnology Inc.) in Tris-buffered saline with 0.1% Tween 20. After washing, the membranes underwent incubation with horseradish peroxidase-conjugated secondary antibodies. The presence of immunoreactive proteins was then detected using enhanced chemiluminescence.

### Statistical analysis

R software (version 4.2.1) was utilized for statistical and data analyses. Continuous data are presented as mean ± standard deviation. The student’s t-test was used to compare two groups for normally distributed variables, whereas the Wilcoxon rank-sum test was employed for non-normally distributed variables. This approach helped to avoid duplicating findings from previous studies. A p-value of less than 0.05 was regarded as statistically difference, following the commonly accepted significance levels. The significance levels were indicated as * for p-values less than 0.05, ** for p-values less than 0.01, and*** for p-values less than 0.001, respectively.

## Results

### Identification of cuproptosis-related genes involved in HIRI

**Figure 2:**
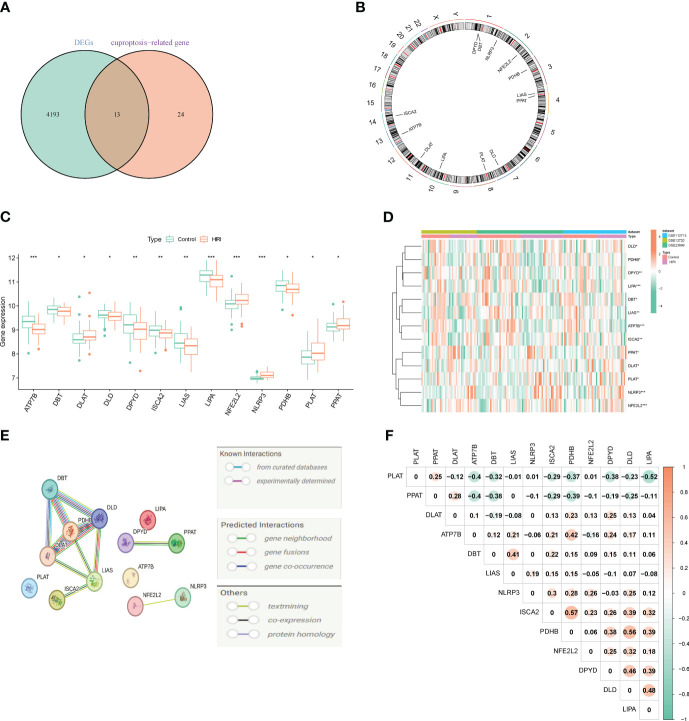
Identifying genes associated with cuproptosis and exhibiting differential expression in HIRI. **(A)** Venn diagram exhibiting the overlap of genes between DEGs and CRGs. **(B)** The locations of the 13 DE-CRGs on 23 chromosomes. **(C)** Boxplots displaying the differential expression of CRGs between the HIRI and control samples. **(D)** Heatmap showing the expression patterns of the 13 DE-CRGs. **(E)** Gene relationship network diagram of the 13 DE-CRGs. **(F)** Correlation analysis of the 13 DE-CRGs, where orange represent positive, and green colors represent negative correlations, respectively. The p values are showed as follows: *, *p* < 0.05; **, *p* < 0.01; ***, *p* < 0.001. CRGs, cuproptosis-related genes. DEG, differential expression genes; DE-CRGs, differentially expressed cuproptosis-related genes.

Merging and batch-normalizing three datasets (GSE12720, GSE23649, and GSE112713), comprising 75 HIRI samples and 80 control samples, yielded a comprehensive dataset. Utilizing the “limma” package with p < 0.05, we identified 4,206 DEGs, with 2,319 downregulated and 1,887 upregulated genes. The gene expression patterns of these DEGs are visually represented in the heatmap (**Supplementary Figure 1**). GO and KEGG analyses of the total DEGs are illustrated in **Supplementary Figure 2**. By intersecting the 4,260 DEGs with 37 CRGs, we pinpointed 13 DE-CRGs (ATP7B, DBT, DLAT, DLD, DPYD, ISCA2, LIAS, LIPA, NFE2L2, NLRP3, PDHB, PLAT, PPAT) exhibiting significant differences between HIRI and control groups (**Figure 2A**). Mapping the chromosomal locations of these 13 DE-CRGs on a loop graph is shown in **Figure 2B**. Notably, DLAT, NFE2L2, NLRP3, PLAT, and PPAT were upregulated in HIRI, whereas ATP7B, DBT, DLD, DPYD, ISCA2, LIAS, LIPA, and PDHB were downregulated (**Figures 2C, D**). To explore potential crosstalk among these 13 DE-CRGs, PPI analyses were conducted using STRING, as depicted in **Figure 2E**. The correlations between the 13 DE-CRGs are depicted in **Figure 2F**. ATP7B was positively associated with DBT, LIAS, ISCA2, DPYD, DLD, LIPA, and NLRP3 was negatively correlated with DYPD.

### Enrichment analysis of the differential CRGs

**Figure 3:**
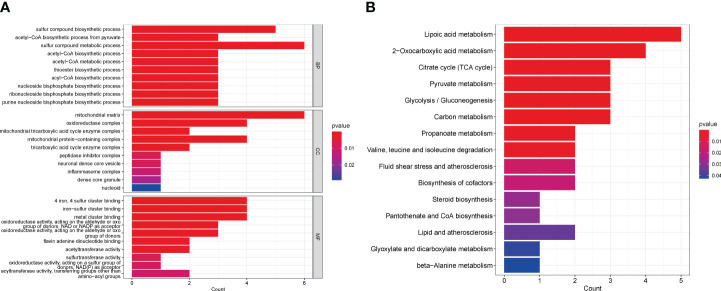
Functional analyses of DE-CRGs. **(A)** Bar charts of the GO enrichment analysis of the 13 DE-CRGs. **(B)** Bar charts of the KEGG enrichment analysis of the 13 DE-CRGs. BP, Biological process; CC, Cellular component; DE-CRGs, differentially expressed cuproptosis-related genes; GO, Gene Ontology; MF, Molecular function; and KEGG, Kyoto Encyclopedia of Genes and Genomes.

Based on these 13 DE-CRGs, we conducted KEGG and GO enrichment analyses to demonstrate the biological functions and pathways utilizing the “ClusterProfler” package. The biological process analysis indicated that it is enriched in sulfur compound metabolic and compound biosynthetic process. Cell component analysis revealed significant involvement in the oxidoreductase complex, mitochondrial matrix, mitochondrial protein-containing complex, and mitochondrial TCA enzyme complex. Molecular Function (MF) analysis highlighted associations with 4-iron, 4-sulfur cluster binding, metal cluster binding, and iron-sulfur cluster binding (**Figure 3A**). Notably, the KEGG pathway analysis unveiled that the 13 DE-CRGs were linked to lipoic acid metabolism, citrate cycle (TCA cycle), 2-oxocarboxylic acid metabolism, pyruvate metabolism, carbon metabolism, and glycolysis/gluconeogenesis (**Figure 3B**).

### Construction of diagnostic marker genes for HIRI

**Figure 4:**
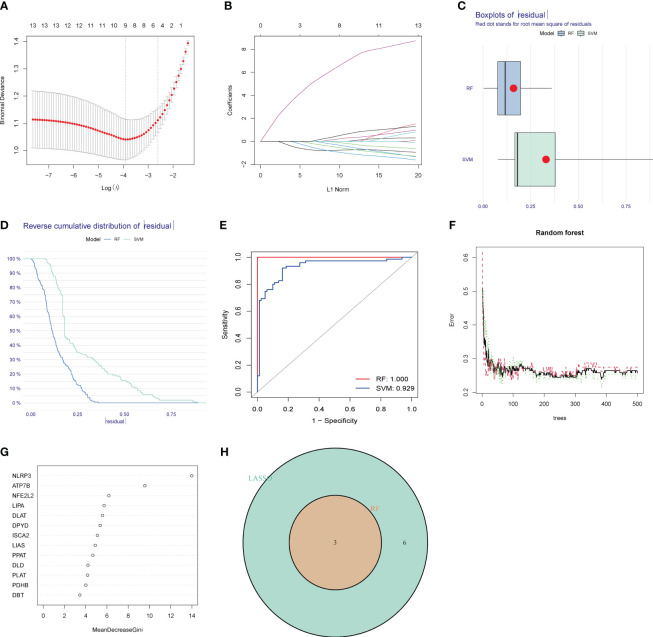
Identification of diagnostic marker genes for HIRI through machine learning. **(A)** LASSO coefficient analysis is depicted using solid vertical lines to represent the partial likelihood of deviation SE. Additionally, a dotted vertical line is drawn at the optimal lambda value. LASSO coefficient analysis. Solid vertical lines represent the partial likelihood of deviation SE. The dotted vertical line is drawn at the optimal lambda. **(B)** The LASSO model undergoes ten-fold cross-validation for adjusted parameter selection. Each curve on the graph represents a specific gene. **(C)** The boxplots and **(D)** the reverse cumulative distribution of residual exhibit the lowest residual distribution between the RF and SVM models. **(E)** The analysis of the overall ROC curve revealed that the RF model had a higher AUC value than the SVM model. **(F)** Correlation between the number of random forest trees and the error rate. The red line signifies the error rate of the HIRI group, the green line represents the error rate of the control group, and the black line denotes the overall sample error rate. **(G)** The rank of genes is based on their relative importance. **(H)** Venn diagram showing the overlap of marker genes between the LASSO, random forest, and SVM RFE algorithms.

To address the individual complexity and heterogeneity among HIRI patients and healthy controls, we employed a combination of statistical methods, including LASSO regression, RF, and SVM–RFE, to identify candidate CRG regulators from the 13 DE-CRGs. This comprehensive approach aimed to enhance the prediction of HIRI diagnosis. Utilizing the LASSO logistic regression algorithm, we successfully identified thirteen DG-CRGs (**Figures 4A, B**). In the analysis of the reverse cumulative distribution of |residual| and boxplots of residual, the RF model consistently demonstrated the lowest residual distribution compared to the SVM model (**Figures 4C, D**). The overall ROC curve revealed that the RF model exhibited a higher AUC value than the SVM model, supporting the selection of the RF model as the most appropriate training model (**Figure 4E**). The relationship between the number of trees in a random forest model and the error rate is depicted in **Figure 4F**. Additionally, we ranked the explanatory variables by importance (**Figure 4G**). The hub genes identified through LASSO, SVM–RFE, and RF were subsequently overlapped using a Venn diagram. Finally, the three key genes (ATP7B, NFE2L2, and NLRP3) were identified for further analysis (**Figure 4H**).

### Validation of marker gene expression

**Figure 5:**
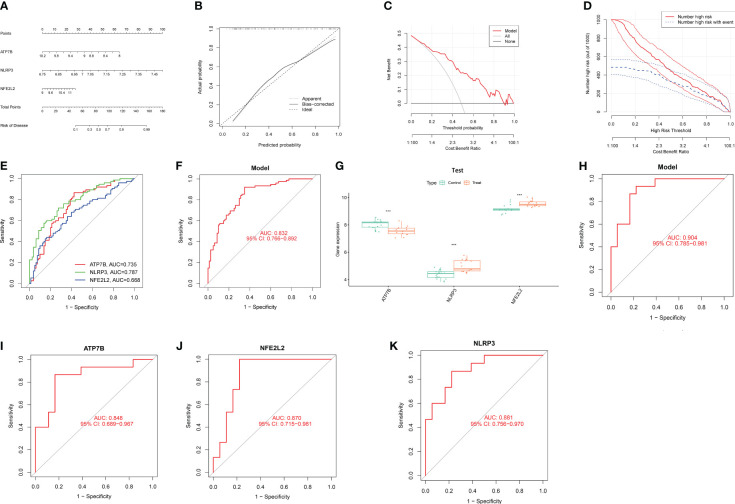
Confirmation of marker gene expression. **(A)** Nomogram graph of the three marker genes. **(B)** Calibration plot showing the diagnostic accuracy of the nomogram model. **(C)** DCA demonstrates the predictive performance of nomogram models. **(D)** The clinical impact curve demonstrates a superior diagnostic capability of the nomogram model. **(E)** The ROC outcomes for the three marker genes. The AUC values for ATP7B, NLRP3, and NFE2L2 were 0.735, 0.787, and 0.668, respectively. **(F)** ROC results of the three-gene-based model based on three-fold cross-validation. The AUC value is 0.832. **(G)** Boxplots demonstrate significant alterations in the expression of three specific differentially expressed CRGs between the HIRI and control samples in the dataset GSE15480 and GSE228782. **(H)** The AUC value of ROC curves for all three hub genes together in GSE15480 and GSE228782. **(I)** The AUC value of the ROC curves for ATP7B. **(J)** The AUC value of the ROC curves for NFE2L2 **(K)** The AUC value of the ROC curves for NLRP3. AUC, area under the curve; DCA, decision curve analysis; and ROC, receiver operating characteristic. ***, p < 0.001.

In order to assess the predictive efficacy of the three central genes, NLRP3, ATP7B and NFE2L2, a nomogram model was constructed using the “rms” package for patients with HIRI (**Figure 5A**). After assigning a score to each biomarker on the nomogram, the risk of HIRI was predicted based on the cumulative score. By analyzing the calibration curves, it was determined that the nomogram model accurately predicted the rate of HIRI positivity (**Figure 5B**). In the context of Decision Curve Analysis (DCA), the nomogram model demonstrated significantly higher net benefits compared to individual hub genes, indicating a high level of accuracy and providing a solid foundation for physician decision-making (**Figure 5C**). The clinical impact curve further illustrated the relatively high diagnostic ability of this nomogram model (**Figure 5D**). To elucidate the predictive value of individual genes, ROC curves for the three marker genes were generated (**Figure 5E**), all of which exhibited values greater than 0.6. The ROC curve analysis collectively showed that the signature of the three genes had a high diagnostic value in HIRI (AUC=0.832, **Figure 5F**). Additionally, the expression and ROC curves of the three hub genes were verified utilizing the GSE15480 and GSE228782 datasets. The results indicated that the expression of ATP7B was downregulated, whereas the expression of NLRP3 and NFE2L2 was upregulated (**Figure 5G**). As showed in **Figure 5H**, the AUC value of ROC curves for all three hub genes together was 0.904, and the AUC values for three individual hub genes were all greater than 0.8 in the GSE15480 and GSE228782 datasets (ATP7B, AUC = 0.848; NFE2L2, AUC = 0.870; and NLRP3, AUC = 0.881) (**Figures 5I–K**), suggesting a very powerful predictive ability for all three hub genes. These findings indicate that the three marker genes could potentially serve as diagnostic biomarkers for HIRI.

### Profile of GSEA and GSVA

**Figure 6:**
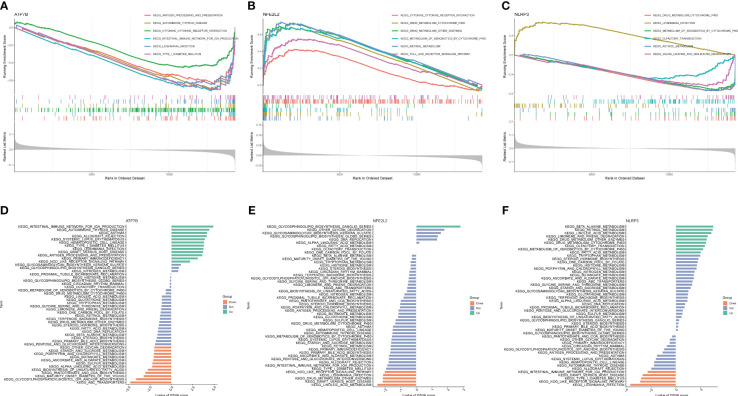
GSEA and GSVA of three marker genes. The GSEA enrichment method was used to conduct KEGG pathway enrichment analyses for **(A)** ATP7B, **(B)** NFE2L2, and **(C)** NLRP3. The visualization is based on the arrangement of enrichment scores, highlighting the two items with the highest and lowest enrichment scores. The visualization highlights the items with the highest and lowest enrichment scores, based on the arrangement of enrichment scores. The KEGG pathway enrichment analyses of **(D)** ATP7B, **(E)** NFE2L2 and **(F)** NLRP3 were performed using the GSVA enrichment method, and the enrichment scores were used to visualize the top 50 pathways.

Based on the KEGG and GO pathways, we conducted single-gene GSEA to identify the primary signaling pathways relevant to this model. The KEGG’s GSEA results indicated that ATP7B and NFE2L2 was involved the interaction between the cytokine and the-cytokine receptor (**Figures 6A–C**). Furthermore, NFE2L2 and NLRP3 were related to drug metabolism by cytochrome P450, xenobiotic metabolism by cytochrome P450 and retinol metabolism (**Figures 6B, C**), while ATP7B and NLRP3 were involved in *leishmania* infection. Furthermore, we found that ATP7B was associated with antigen processing and presentation, autoimmune thyroid disease, type I diabetes mellitus and intestinal immune network for IgA production (**Figure 6A**), while NFE2L2 was involved in the Toll-like receptor signaling pathway (**Figure 6B**). NLRP3 was associated with olfactory transduction, isoleucine degradation, and valine leucine (**Figure 6C**). The GSEA result of GO enrichment is presented in **Supplementary Figure 2**.

Next, GSVA was utilized to identify the differentially active pathways between low- and high-expression subtypes, based on the expression levels of the three hub genes. Our analysis unveiled that ATP7B overexpression activated multiple pathways, including autoimmune thyroid disease, the intestinal immune network for IgA production, asthma, rejection of allografts, systemic lupus erythematosus, hematopoietic cell lineage, type I diabetes mellitus, *leishmania* infection, graft versus host disease, antigen processing and presentation, primary immunodeficiency and Nod receptor signaling pathway (**Figure 6D**). In contrast, low expression of ATP7B was associated with ABC transporters, glycosylphosphatidylinositol GPI anchor biosynthesis, maturity-onset diabetes of the young, pantothenate and COA biosynthesis, biosynthesis of unsaturated fatty acid, alpha-linolenic acid metabolism, sulfur metabolism, ascorbate and aldarate metabolism, butanoate metabolism, porphyrin, and chlorophyll metabolism, starch and sucrose metabolism, and other glycan degradation (**Figure 6D**). The overexpression of NEF2L2 activates the glycosphingolipid biosynthesis ganglio series. Conversely, when NEF2L2 expression is low, it triggers the activation of linoleic acid metabolism and is implicated in graft-versus-host disease, as depicted in Figure 6E. On the other hand, elevated expression of NLRP3 may result in heightened activity of β-alanine metabolism and retinol metabolism. Low NLRP3 expression was associated with *leishmania* infection, graft versus host disease, type I diabetes mellitus, Nod-like receptor signaling pathway, and intestinal immune network for IgA production (**Figure 6F**).

### Landscapes of immune infiltration in HIRI

**Figure 7:**
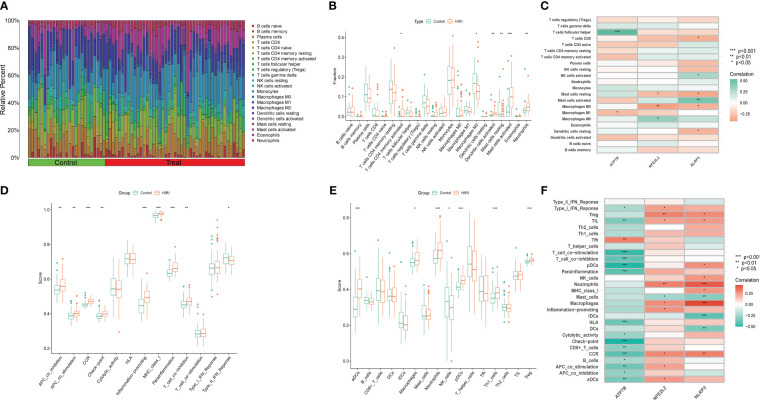
Immune infiltration analysis between HIRI and controls using the CIBERSORT and ssGSEA algorithm. **(A)** The differences in the relative abundances of 22 infiltrated immune cells between the HIRI and control samples. **(B)** The boxplots indicate the variances in immune cell infiltration between the HIRI and control samples. The results showed that 6/22 immune cells were substantially different between the HIRI and control groups. **(C)** The correlation between 22 immune cells and three marker genes. Red and green colors represent positive and negative correlations, respectively. **(D, E)** Boxplots indicate the differences in immune cells and function between the HIRI and control samples. **(F)** The correlation between 29 immune cells and functions and three marker genes. Red and green colors represent positive and negative correlations, respectively. The p-values were showed as: *, *p* < 0.05; **, *p* < 0.01; ***, *p* < 0.001.

HIRI is characterized by the infiltration of immune cells into hepatic plaques and lobules. Notably, cuproptosis has also been identified as a regulator in inflammation modulation. To investigate whether cuproptosis could contribute to the progression of HIRI by mediating immune infiltration, we utilized algorithms and single-sample gene set enrichment analysis (ssGSEA). Initially, the CIBERSORT algorithm was applied to evaluate differences in the immune microenvironment between HIRI and control samples. **Figure 7A** illustrates the proportion of 22 different immune cells’ expression between the HIRI and control samples. **Figure 7B** illustrates the expression of six types of immune cells that exhibit a significant difference between the two groups. Specifically, we found that compared with controls, activated CD4 memory T cells, activated dendritic cells, activated mast cells, and neutrophils were more abundant in HIRI patients, while M2 macrophages and resting mast cells were less abundant (**Figure 7B**). The correlation analysis revealed a positive correlation between ATP7B and follicular helper T cells and a negative correlation with M1 macrophage (**Figure 7C** and **Table S2**). Both NFE2L2 and NLRP3 were negatively correlated with resting mast cells and the M2 macrophage. These results suggest that modifications in the immune microenvironment may contribute to the development of HIRI.

Next, we used the ssGSEA algorithm to analyze the enrichment scores of different immune cells and functions or pathways between the HIRI and control groups. For immune functions, the scores of APC-co-stimulation, APC-co-inhibition, CCR, MHC class I, checkpoint inflammation-promoting, para-inflammation and T cell co-inhibition were higher in HIRI than in the control group (**Figure 7D**). We also found that activated neutrophils, dendritic cells, plasmacytoid dendritic cells, macrophages, TH1 cells and Treg were significantly upregulated in HIRI patients (**Figure 7E**). The results also showed that ATP7B was significantly associated with type I interferon response, T cell co-stimulation, T cell co-inhibition, plasmacytoid dendritic cells, para-inflammation, checkpoint signaling, and CCR (**Figure 7F**). Together, these findings further affirm the correlation of the three hub genes with the immune infiltration microenvironment.

### Identification of drug candidates

**Figure 8:**
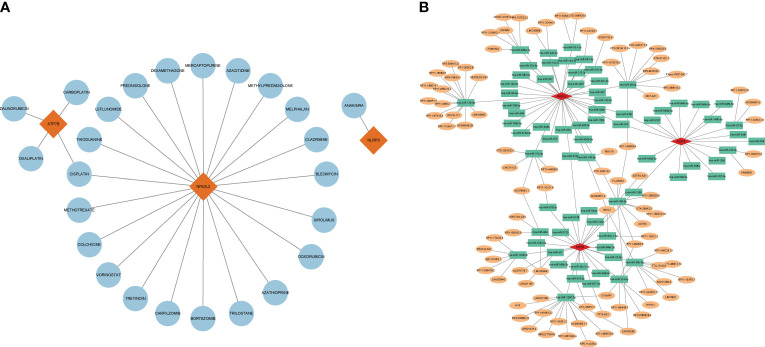
Gene-targeted drugs and ceRNA networks. **(A)** Network of interaction of mRNA drugs. The blue circular node denotes the drugs. **(B)** The ceRNA network is based on marker genes. Pink dots represent mRNA, green dots represent miRNA, and orange dots represent lncRNA.

For advancing future treatments of HIRI, we examined the interaction between hub genes and drugs using the DGIdb database. Cytoscape analysis illustrated the interaction between gene markers and drugs (refer to **Figure 8A**). Totally, 25 gene-targeted drugs were identified: 21 for NFE2L2, 4 for ATP7B, and 1 for NLRP3. Notably, NFE2L2 and ATP7B share a common drug target, cisplatin.

### CeRNA networks based on marker genes

Using the TargetScan, miRanda, and miRDB databases, a ceRNA network was developed based on the three marker genes. A total of three mRNAs, 72 miRNAs, and 92 lncRNAs were identified (**Figure 8B**). The results indicated that 32 lncRNAs could regulate the expression of ATP7B through competitive binding of hsa-miR-1207-5p, hsa-miR-484, hsa-miR-421, hsa-miR-1238-3p, and sa-miR-30b-3p. Out of these, 15 shared lncRNAs were found to target hsa-miR-1207-5p. In total, 46 lncRNAs were able to competitively bind to 36 miRNAs, including hsa-miR-129-5p, hsa-miR-499a-3p, hsa-miR-16-1-3p, hsa-miR-28-5p, hsa-miR-27a-3p, and subsequently regulated NFE2L2. Among them, 14 and 9 lncRNAs target hsa-miR-28-5p and hsa-miR-129-5p, respectively. For NLRP3, there were a total of 18 regulated miRNAs, including hsa-miR-22-3p and hsa-miR-223-3p.

### Altered expression of CRGs in HIRI

**Figure 9:**
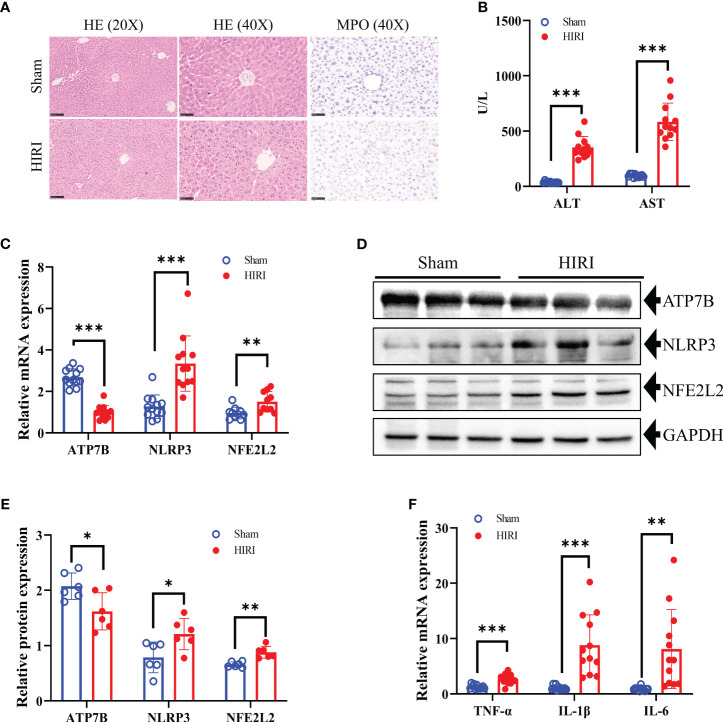
Construction of the HIRI mice model and detection the expression of hub CRGs in HIRI. **(A)** Representative HE and MPO staining of the liver tissue from mice subjected to sham or HIRI. **(B)** ALT and AST values in both sham and HIRI mice (n = 12 per group). **(C)** The mRNA levels of ATP7B, NLRP3, and NFE2L2 in liver samples from sham and HIRI mice using RT-PCR (n = 10-12 per group). **(D, E)** Protein expression levels of ATP7B, NLRP3, and NFE2L2 in the liver of sham and HIRI mice. GAPDH served as the loading control (n = 6 per group). **(F)** Pro-inflammatory cytokine mRNA levels (TNF-α, IL-1, and IL-6) in liver samples from sham and HIRI mice (n = 12 per group). *, *p* < 0.05; **, *p* < 0.01; and ***, *p* < 0.001. ALT, serum alanine aminotransferase; AST, aspartate aminotransferase.

After 30 minutes of hepatic ischemia and 6 hours of reperfusion, liver function and histopathology were evaluated. The success of the HIRI model was confirmed by liver histopathology. H&E staining also demonstrated pathological damage to the liver tissues of HIRI mice (**Figure 9A**). Furthermore, the infiltration of MPO-positive neutrophil cells was increased by HIRI compared with their numbers in the sham (**Figure 9A**). In addition, serum ALT and AST levels were raised significantly in HIRI mice compared with baseline or with sham operated mice (**Figure 9B**). To further investigate the role of the CRGs in HIRI, the hepatic mRNA and protein levels of three hub genes ATP7B, NLRP3 and NFE2L2 were significantly altered in the HIRI mice compared with the control mice (**Figures 9C–E**). The mRNA levels of the inflammatory cytokines TNF-α, IL-1, and IL-6 were significantly elevated in HIRI mice compared with sham mice (**Figure 9F**). These findings imply that CRGs have a significant impact on the development of HIRI, further confirming their potential role in regulating the progression of HIRI.

## Discussion

As a typical complication following liver surgery such as partial hepatectomy and liver transplantation, HIRI often results in a poor postoperative prognosis, extended hospitalization, and increased medical expenses. Mechanically, the ischemia and reperfusion process is greatly influenced by mitochondria activities by initiating oxidative stress and inflammatory injuries (16–18). Ischemia and reperfusion-induced hepatocyte death is characterized by various distinct, often complementary or overlapping, regulated form of cell death, including necrosis, necroptosis, pyroptosis, ferroptosis, and apoptosis (19). Clarifying the mechanisms underlying hepatocyte death and exploring effective intervention strategies have always been paramount in HIRI research. Cuproptosis, a newly identified form of cell death dependent on mitochondrial respiration, has been implicated to play critical roles in ischemia-reperfusion damages to the heart and brain (20–22). Therefore, it is necessary to investigate the mechanism, and the signaling pathways of cuproptosis to identify new treatment targets for HIRI.

In this study, we examined the differential expression of CRGs in both HIRI and control liver samples obtained from the GEO database. As a result, we identified 13 DE-CRGs (ATP7B, DBT, DLAT, DLD, DPYD, ISCA2, LIAS, LIPA, NFE2L2, NLRP3, PDHB, PLAT, and PPAT) related to cuproptosis. At the protein level, pairs of DE-CRGs such as DBT-OLD-PDHE-DLAT-LIAS, NFE2L2-NLRP3, DPYD-PPAT, and lipoic acid synthetase (LIAS)-ISCA2 were closely related, indicating that there are diverse interactions between CRGs at the gene and protein levels during the development of HIRI. GO and KEGG enrichment analyses revealed that the identified CRGs were significantly involved in the TCA pathway and lipoic acid metabolism. This aligns with the earlier findings that cuproptosis is triggered by disruption of the TCA cycle when copper binds to lipoylated elements (9), At the same time, LIAS is recognized as contributing to the creation of metabolic enzymes linked to mitochondria, essential for energy generation and defense against oxidants (23). Intriguingly, our data indicated no discernible difference in the related cuproptosis regulator, FDX1 and LIAS, between the HIRI and control liver samples. Therefore, further studies are imperative to delineate the intricate nexus between CRGs, the TCA cycle and lipoic acid metabolism in the context of HIRI. Additionally, the Molecular function section in the GO analysis highlighted that the identified 13DE-CRGs were associated with the binding of the iron-sulfur cluster, reflecting previous research that the loss of iron-sulfur cluster proteins was part of the onset of cuproptosis (9). In subsequent procedures, we used three machine learning paradigms (LASSO, RF, and SVM-REE) and finally identified three central genes (NLRP3, ATP7B, and NFE2L2) that accurately predict the risk of HIRI with an AUC value of 0.832. Furthermore, the external validation database further confirmed its reliability (AUC= 0.904). In the validation database, ATP7B, NFE2L2, and NLRP3 exhibited AUC values exceeding 0.8. In addition, a nomogram model, calibration curves, and DCA were constructed using ATP7B, NFE2L2, and NLRP3. This further validated the diagnostic predictive efficacy and clinical value of the model. Therefore, we conclude that the three-gene model is a reliable and robust biomarker to predict the diagnosis of HIRI.

The sterile inflammatory response of HIRI was diven by innate immunity initiated by recognizing pattern-recognition receptors (PRRs), including NLRP3 (2, 24, 25). NLRP3 has been identified as a major contributor to liver injury and amplification of liver inflammation, making it a promising therapeutic target. In the LIRI mouse model, NLRP3 knockout protected the liver from I/R injury and attenuated the inflammatory response by reducing the release of proinflammatory cytokines including IL-1β, IL-18, TNF-α, and IL-6. Some studies also discovered that NLRP3 is downregulated in stellate cells and hepatocytes but strongly expressed in macrophages and monocytes, suggesting that macrophages are the primary effectors of NLRP3 (25, 26). Moreover, NLRP3 inflammasome-induced pyroptosis has been established to contribute significantly to the progression of HIRI (27). Nonetheless, the relationship between NLRP3 and cuproptosis in HIRI remains elusive. Further investigations using GSEA and GSVA in our research indicated that the participation of NLRP3 in drug metabolism via cytochrome P450, linoleic acid metabolism, beta-alanine metabolism, and fatty acid metabolism could provide potential insights. The copper chaperone ATP7B is a hepato-specific Golgi-located ATPase which loads copper into the serum copper-protein ceruloplasmin during its production and also transports excess copper into the bile through an intricate trafficking pathway, ensuring copper homeostasis. Wilson’s disease (WD) is a well-known inborn mistake of copper metabolism, is linked to impaired performance of ATP7B (5). Previous studies have predominantly concentrated ATP7B’s function in copper metabolism disorders or elevated resistance to platinum-based chemotherapy such as Wilson’s disease and, its involvement in Alzheimer’s disease and cancer (28–30). However, limited research has explored ATP7B’s role in HIRI. In our study, we observed lower expression of ATP7B in the HIRI mice compared to the normal. Furthermore, both ATP7B mRNA and protein levels exhibited a reduction in HIRI mice when compared to the control group. Furthermore, GSEA analysis unveiled that the involvement of ATP7B in the cytokine–cytokine receptor interaction, antigen processing and presentation. Additional GSVA results demonstrated that low expression of ATP7B was involved in the process of energy metabolism, such as ABC (ATP-binding cassette transporter) transporters, pantothenate and enzyme A biosynthesis, unsaturated fatty acid biosynthesis, sulfur metabolism, starch and sucrose metabolism, and other glycan degradation. It is well documented that ATP7B is intertwined with lipid metabolism and can damage crucial cellular organelles for ROS energy metabolism during the development of Wilson’s disease (31, 32). Excess ROS production can inhibit other antioxidant defense mechanisms in HIRI, leading to increased oxidative damage (33, 34). Thus, further exploration of the role of ATP7B in the pathogenesis of HIRI is necessary for future detailed insights. NFE2L2, or Nrf2, functions as a transcription factor in antioxidant defense and detoxification (35). In HIRI, ROS generation leads to membrane damage and oxidative stress. Activating the Nrf2 transcription factor enhances downstream antioxidant, and serving as a primary cellular defense against cytotoxic effects of oxidative stress and promoting hepatic recovery (36). In our study, we observed higher NEF2L2 expression in HIRI samples than in control livers. Both mRNA and protein levels of NFE2L2 increased in HIRI mice, consistent with previously reported findings (37). However, some studies have yielded contradictory results (38, 39). GSEA and GSVA revealed NFE2L2 involvement in immune processes such as the cytokine–cytokine receptor interaction pathway and the Toll-like receptor signaling pathway. On the contrary, previous studies suggested NFE2L2 regulates inflammation through HO-1, NF-κB, and NLRP3 pathways during HIRI (37, 38). Crosstalk between Nrf2 and NLRP3 inflammasome shows Nrf2 activation represses NLRP3 inflammasome and inflammation (40). ROS link both pathways, asNrf2 activation detoxify ROS, whereas ROS are believed to activate the NLRP3 inflammasome (26, 41). Additionally, both ATP7B and NFE2L2 were associated with the cytokine and cytokine receptor interaction pathway, we observed significantly elevated expression of associated cytokines TNF-α, IL-1, and IL-6 in HIRI mice (**Figure 9F**). These cumulative results indicate the pivotal and multifaceted function of the three identified hub genes in the development of HIRI. Elucidating the underlying mechanisms of these genes and cuproptosis is crucial to advance our understanding and therapeutic strategies for this pathological condition.

The cellular mechanisms that govern HIRI are complex, and multiple immune cells are involved in its pathogenesis. Activating inflammatory signals exacerbates HIRI by recruiting macrophages, neutrophils, cytotoxic T cells, and dendritic cells activating NK and NKT cells (2, 42). In our study, we employed the CIBERSORT algorithm and ssGSEA to analyze immune infiltration in HIRI. The ratios of M2 macrophages and resting mast cells were lower in liver samples from HIRI patients than in control liver samples, while activated CD4 memory T cells, dendritic cells (DCs), activated mast cells (MCs), and neutrophils were higher in the HIRI group than in the control group. Macrophages play a crucial role in initiating the immune response during HIRI, they detect early signs of organ damage through PRR, become activated, and secret chemokines and cytokines, recruiting various immune cells including T cells, neutrophils, and monocytes, in circulation, thus exacerbating hepatocellular damage (2, 43). In the polarization of liver macrophages, M1 macrophages predominate during ischemia/reperfusion, producing a range of pro-inflammatory cytokines that intensify the damage and recruit other immune cells. Conversely, M2 macrophages can ameliorate ischemia/reperfusion by increasing anti-inflammatory factors (44). CD4+ T cells are also acknowledged as crucial mediators of inflammation in the HIRI cascade (45, 46). These cells, once activated, can enhance macrophage activation, magnify their effect, and initiate an immune cascade. Inhibition of T cell immunoglobulin mucin-1 in CD4+ T cells has been reported to diminish macrophage activation and reduce HIRI (47). Therapeutic strategies aimed at CD4+ T cells may attenuate hepatic IRI, showcasing substantial therapeutic promise. The role of DCs in HIRI is complex, as depletion of DCs in CD11c-diphtheria toxin receptor (DTR) transgenic mice leads to an aggravation of liver damage. Conversely, the adoptive transfer of conventional DCs can reduce this injury (48). However, increasing the number of DCs with exogenous GM-CSF aggravated liver injury via necrotic cell-released high mobility group 1 (HMGB1) and TLR4 activation (49). The complexity of the role played by DCs may be due to the different roles of their various subtypes, which also indicates possible directions for our future research. During HIRI, neutrophil accumulation and activation also contribute much to hepatocellular damage (2), and we also observed neutrophil accumulation through liver MPO staining during mice HIRI (**Figure 9A**). It has also been reported that activated neutrophils release neutrophil extracellular traps to enhance liver injury further (50). MCs possess a distinctive distribution surrounding the microvasculature. They may serve as primary responders to initial or particular aspects of ischemia/reperfusion pathogenesis by discharging pre-stored mediators from their granules (51). Throughout the HIRI process, MCs accumulate and emit various bioactive substances, thereby modifying the local tissue microenvironment and influencing the inflammatory reaction. Additional research is needed to comprehensively understand their effect on the pathophysiology of HIRI (52, 53). Furthermore, our results suggest a close correlation between NLRP3 and various immune cells and functions, aligning with prior research (54, 55). These findings underscore the significant involvement of diverse immune cells in HIRI progression; enhancing insight into this intricate and constantly evolving process could facilitate the development of innovative therapeutic strategies and improve patient outcomes.

Given the promising potential of cuproptosis-targeted therapeutic agents, our research investigated gene-targeted drugs focusing on the three hub genes. Notably cisplatin, a metal-based anticancer drug, targets both ATP7B and NFE2L2 among hub genes, offers hepatoprotective benefits and exerting anti-inflammatory effects (56). Research has shown that non-toxic doses of cisplatin protect mice from ischemia and reperfusion damage by inhibiting the release of HMGB1 and modulating cellular survival and stress signaling pathways, specifically through induction of autophagy and activation of mitogen-activated protein kinase (57). Long noncoding RNAs (lncRNAs), serving as compete endogenous RNAs, can vie for miRNA binding, thereby regulating mRNA expression and altering the physiological functions of various cells. Considering the possible significance of the lncRNA–miRNA–mRNA pathway, we constructed a relevant ceRNA network for HIRI. Our study indicates that lncRNAs can influence the expression of three CRGs, NLRP3, ATP7B and NFE2L2. This introduces a fresh perspective for investigating drug selection and understanding HIRI pathogenesis through gene-targeted therapeutics and analysis of ceRNA networks. However, the absence of comprehensive in vitro and in vivo studies requires further empirical validation to confirm these findings.

However, our study has its limitations. Primarily, the reliance on data from the GEO public database may introduce selection bias due to the unavailability of raw sequencing data. Furthermore, while mouse models provide some corroboration for our bioinformatics analyses, the scarcity of clinical HIRI samples within our study’s timeframe is a constraint, and additional clinical samples are imperative for elucidating the mechanisms of cuproptosis in HIRI. Furthermore, discrepancies observed between RNA sequences and qRT-PCR results suggest a complex regulatory mechanism of cuproptosis in HIRI, which we could not fully investigate within the temporal scope of this study. Therefore, future research is essential to elucidate this regulatory mechanism further.

## Conclusions

Our investigation reveals a link between CRGs and immune cell infiltration, underscoring the marked variability of the immune response between HIRI patients and healthy liver samples. We employed a machine learning approach to discern a signature based on three CRGs that adeptly identify HIRI patients. These insights contribute to a better understanding of the involvement of cuproptosis in HIRI and its underlying pathological mechanisms, pointing to potential targets for therapeutic intervention.

## Data availability statement

The datasets presented in this study can be found in online repositories. The names of the repository/repositories and accession number(s) can be found in the article/**Supplementary Material**.

## Ethics statement

The animal study was approved by Animal Care and Use Committee of Guangxi Medical University. The study was conducted in accordance with the local legislation and institutional requirements.

## Author contributions

FX: Writing – review & editing, Writing – original draft, Data curation. GH: Writing – original draft, Writing – review & editing, Validation, Data curation. GY: Funding acquisition, Writing – review & editing. SL: Validation, Writing – review & editing. YW: Software, Writing – review & editing. ZT: Writing – review & editing. ZL: Writing – review & editing. ST: Writing – review & editing. SH: Writing – review & editing, Project administration, Funding acquisition. GO: Writing – review & editing, Methodology, Data curation. YZ: Writing – review & editing, Funding acquisition.
