# Supplementary material for: Identification and validation of potential diagnostic signature and immune cell infiltration for HIRI based on cuproptosis-related genes through bioinformatics analysis and machine learning
Source: Front Immunol. 2024 Apr 16;15:1372441. doi: 10.3389/fimmu.2024.1372441 (PMC11058647; doi:10.3389/fimmu.2024.1372441)
Supplement: Supplementary file 1 [file DataSheet_1.docx]

**­­** **Identification and validation of potential diagnostic signature and immune cell infiltration for HIRI based on cuproptosis-related genes through bioinformatics analysis and machine learning**

Fang Xiao^1,2,3,#^, Guozhen Huang^1,2,3,#^, [Guandou Yuan](https://pubmed-ncbi-nlm-nih-gov-443--bjmu.jitui.me/?term=Yuan+G&cauthor_id=35069557)^1,2,3^, Shuangjiang Li^1,2,3,^, Yong Wang^1,2,3^, Zhi Tan^1,2,3^, Zhipeng Liu^1,2,3,^, Stephen Tomlinson^4^, Songqing He^1,2,3^*, Guoqing Ouyang^1,2,3^*, Yonglian Zeng^1,2,3^*

^1^Division of Hepatobiliary Surgery, The First Affiliated Hospital of Guangxi Medical University, Nanning, Guangxi 530021, China.

^2^Key Laboratory of Early Prevention and Treatment for Regional High Frequency Tumor (Guangxi Medical University), Ministry of Education, Nanning, Guangxi 530021, China.

^3^Guangxi Key Laboratory of Immunology and Metabolism for Liver Diseases, Nanning, Guangxi 530021, China.

^4^Department of Microbiology and Immunology, Medical University of South Carolina, Charleston, SC, United States.

^#^ These authors have contributed equally to this work

*Correspondence to:

Yonglian Zeng, Guangxi Key Laboratory of Immunology and Metabolism for Liver Diseases, The First Affiliated Hospital of Guangxi Medical University, NO 6 Shuangyong Road, Nanning 530021, Guangxi, China. E-mail: zyl-lian@163.com

Guoqing Ouyang, Division of Hepatobiliary Surgery, The First Affiliated Hospital of Guangxi Medical University, NO 6 Shuangyong Road, Nanning 530021, Guangxi, China. Email: [Ouyangguoqing@stu.gxmu.edu.cn](mailto:Ouyangguoqing@stu.gxmu.edu.cn).

Songqing He, Division of Hepatobiliary Surgery, The First Affiliated Hospital of Guangxi Medical University, NO 6 Shuangyong Road, Nanning 530021, Guangxi, China. Email: [dr_hesongqing@163.com](mailto:dr_hesongqing@163.com)

**Contents**

[**Supplementary tables** 3](#_Toc155370586)

[**Table S1** the primers of GAPDH, ATP7B, NFE2L2, and NLRP3 3](#_Toc155370587)

[**Table S2** The correlation between 3 cuproptosis genes and immune cells 4](#_Toc155370588)

[**Supplementary Figures** 6](#_Toc155370589)

[**Figure S1** 6](#_Toc155370590)

[**Figure S2** 7](#_Toc155370591)

**Supplementary tables**

**Table S1** the primers of GAPDH, ATP7B, NFE2L2, and NLRP3

| Genes | | Sequences (5’-3’) |
| --- | --- | --- |
| GAPDH | Forward | CCCACTAACATCAAATGGGG |
|  | Reverse | CCTTCCACAATGCCAAAGTT |
| ATP7B | Forward | AAGAGGCCAGTCGGAAAATCT |
|  | Reverse | TTGTCGAAGGCGAAGCTCTG |
| NFE2L2 | Forward | AGAGTGATGGTTGCCCACTT |
|  | Reverse | ATCACACACTTTCTGCGTGC |
| NLRP3 | Forward | AGGCTGCTATCTGGAGGAACTT |
|  | Reverse | TGCAACGGACACTCGTCATC |

**Table S3** The correlation between 3 cuproptosis genes and immune cells

| **Genes** | **Immune cells** | **Correlation** | **P value** |
| --- | --- | --- | --- |
| ATP7B | aDCs | -0.3577 | 0.001733 |
| NLRP3 | aDCs | 0.165974 | 0.154427 |
| NFE2L2 | aDCs | 0.252534 | 0.028828 |
| ATP7B | APC_co_inhibition | -0.29218 | 0.011214 |
| NLRP3 | APC_co_inhibition | -0.14723 | 0.207032 |
| NFE2L2 | APC_co_inhibition | 0.116288 | 0.320444 |
| ATP7B | APC_co_stimulation | -0.37186 | 0.001099 |
| NLRP3 | APC_co_stimulation | 0.009787 | 0.933519 |
| NFE2L2 | APC_co_stimulation | 0.230514 | 0.046628 |
| ATP7B | B_cells | -0.2425 | 0.036302 |
| NLRP3 | B_cells | -0.04213 | 0.719109 |
| NFE2L2 | B_cells | 0.171168 | 0.142018 |
| ATP7B | CCR | -0.4299 | 0.000137 |
| NLRP3 | CCR | 0.307624 | 0.00747 |
| NFE2L2 | CCR | 0.286133 | 0.012824 |
| ATP7B | CD8+_T_cells | -0.29919 | 0.009118 |
| NLRP3 | CD8+_T_cells | -0.00407 | 0.972366 |
| NFE2L2 | CD8+_T_cells | 0.117599 | 0.314984 |
| ATP7B | Check-point | -0.50381 | 5.55E-06 |
| NLRP3 | Check-point | 0.0633 | 0.588774 |
| NFE2L2 | Check-point | 0.11158 | 0.34056 |
| ATP7B | Cytolytic_activity | -0.27531 | 0.017086 |
| NLRP3 | Cytolytic_activity | 0.175903 | 0.130978 |
| NFE2L2 | Cytolytic_activity | 0.016088 | 0.891033 |
| ATP7B | DCs | -0.16549 | 0.155644 |
| NLRP3 | DCs | -0.34819 | 0.002327 |
| NFE2L2 | DCs | 0.088578 | 0.449822 |
| ATP7B | HLA | -0.38068 | 0.000819 |
| NLRP3 | HLA | 0.130156 | 0.265084 |
| NFE2L2 | HLA | 0.058023 | 0.620973 |
| ATP7B | iDCs | -0.11007 | 0.346402 |
| NLRP3 | iDCs | -0.42364 | 0.000175 |
| NFE2L2 | iDCs | -0.10212 | 0.383309 |
| ATP7B | Inflammation-promoting | -0.22626 | 0.051136 |
| NLRP3 | Inflammation-promoting | 0.170811 | 0.14264 |
| NFE2L2 | Inflammation-promoting | 0.266645 | 0.020752 |
| ATP7B | Macrophages | -0.21502 | 0.064076 |
| NLRP3 | Macrophages | 0.437667 | 0.000101 |
| NFE2L2 | Macrophages | 0.277882 | 0.015784 |
| ATP7B | Mast_cells | -0.15898 | 0.172751 |
| NLRP3 | Mast_cells | -0.32114 | 0.005148 |
| NFE2L2 | Mast_cells | -0.2494 | 0.030939 |
| ATP7B | MHC_class_I | -0.17866 | 0.12497 |
| NLRP3 | MHC_class_I | 0.256046 | 0.026868 |
| NFE2L2 | MHC_class_I | -0.01135 | 0.922998 |
| ATP7B | Neutrophils | -0.10569 | 0.366024 |
| NLRP3 | Neutrophils | 0.403073 | 0.000375 |
| NFE2L2 | Neutrophils | 0.305009 | 0.007795 |
| ATP7B | NK_cells | -0.00872 | 0.940812 |
| NLRP3 | NK_cells | 0.256104 | 0.026568 |
| NFE2L2 | NK_cells | 0.111111 | 0.342605 |
| ATP7B | Parainflammation | -0.4177 | 0.000219 |
| NLRP3 | Parainflammation | 0.130868 | 0.262465 |
| NFE2L2 | Parainflammation | 0.201282 | 0.083337 |
| ATP7B | pDCs | -0.493 | 9.27E-06 |
| NLRP3 | pDCs | 0.269502 | 0.019644 |
| NFE2L2 | pDCs | 0.040655 | 0.72911 |
| ATP7B | T_cell_co-inhibition | -0.40666 | 0.00033 |
| NLRP3 | T_cell_co-inhibition | -0.00481 | 0.96736 |
| NFE2L2 | T_cell_co-inhibition | 0.024253 | 0.836367 |
| ATP7B | T_cell_co-stimulation | -0.47095 | 2.51E-05 |
| NLRP3 | T_cell_co-stimulation | 0.018321 | 0.875812 |
| NFE2L2 | T_cell_co-stimulation | 0.056003 | 0.633198 |
| ATP7B | T_helper_cells | -0.1528 | 0.190605 |
| NLRP3 | T_helper_cells | -0.10239 | 0.382045 |
| NFE2L2 | T_helper_cells | 0.136545 | 0.242752 |
| ATP7B | Tfh | 0.306572 | 0.007685 |
| NLRP3 | Tfh | -0.17664 | 0.129347 |
| NFE2L2 | Tfh | 0.121253 | 0.300076 |
| ATP7B | Th1_cells | -0.22179 | 0.055995 |
| NLRP3 | Th1_cells | 0.194851 | 0.093889 |
| NFE2L2 | Th1_cells | 0.166459 | 0.15348 |
| ATP7B | Th2_cells | -0.22708 | 0.050278 |
| NLRP3 | Th2_cells | 0.202873 | 0.080933 |
| NFE2L2 | Th2_cells | 0.006999 | 0.95248 |
| ATP7B | TIL | -0.34868 | 0.002293 |
| NLRP3 | TIL | 0.242418 | 0.036369 |
| NFE2L2 | TIL | 0.231766 | 0.045417 |
| ATP7B | Treg | -0.17053 | 0.143314 |
| NLRP3 | Treg | 0.284239 | 0.013711 |
| NFE2L2 | Treg | 0.30454 | 0.007895 |
| ATP7B | Type_I_IFN_Reponse | -0.23425 | 0.043309 |
| NLRP3 | Type_I_IFN_Reponse | 0.194936 | 0.093743 |
| NFE2L2 | Type_I_IFN_Reponse | 0.238024 | 0.039745 |
| ATP7B | Type_II_IFN_Reponse | -0.03681 | 0.75337 |
| NLRP3 | Type_II_IFN_Reponse | -0.11727 | 0.315628 |
| NFE2L2 | Type_II_IFN_Reponse | 0.04431 | 0.705817 |

**Supplementary Figures**

**Figure S1**


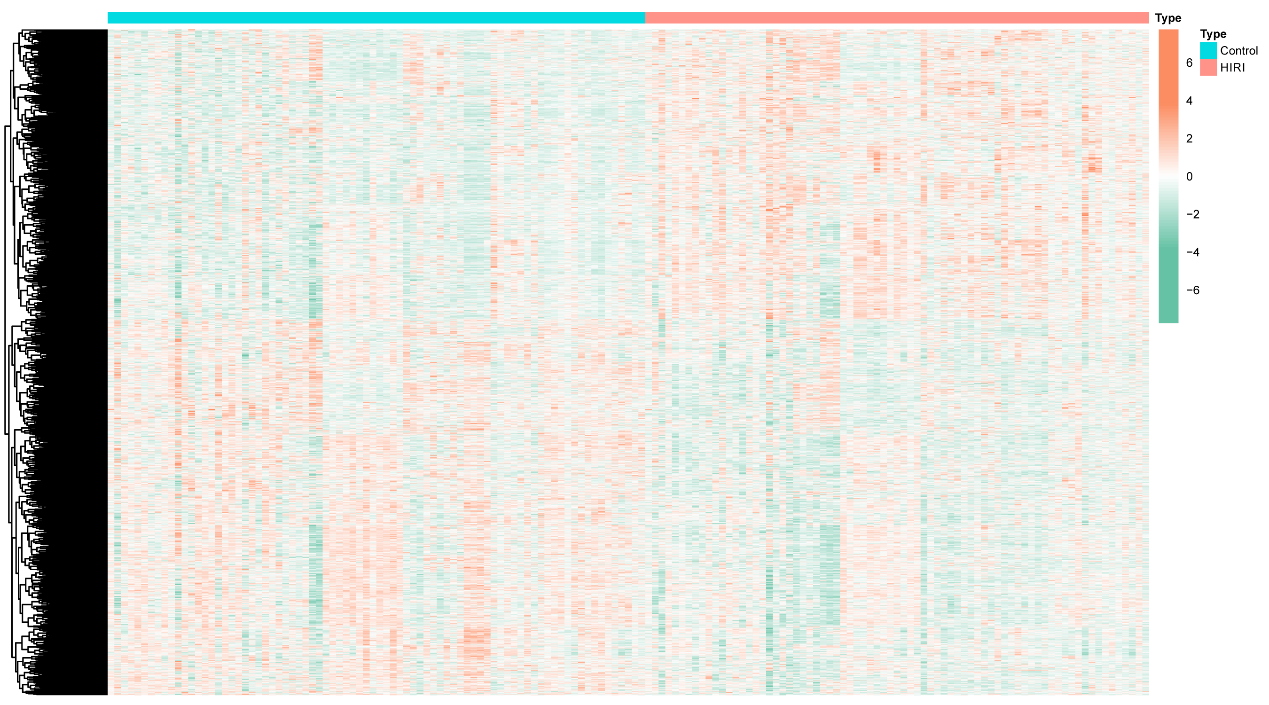


The expression patterns of 4,206 DEGs were presented in the heatmap between HIRI and control. DEG: differential expression genes.

**Figure S2**


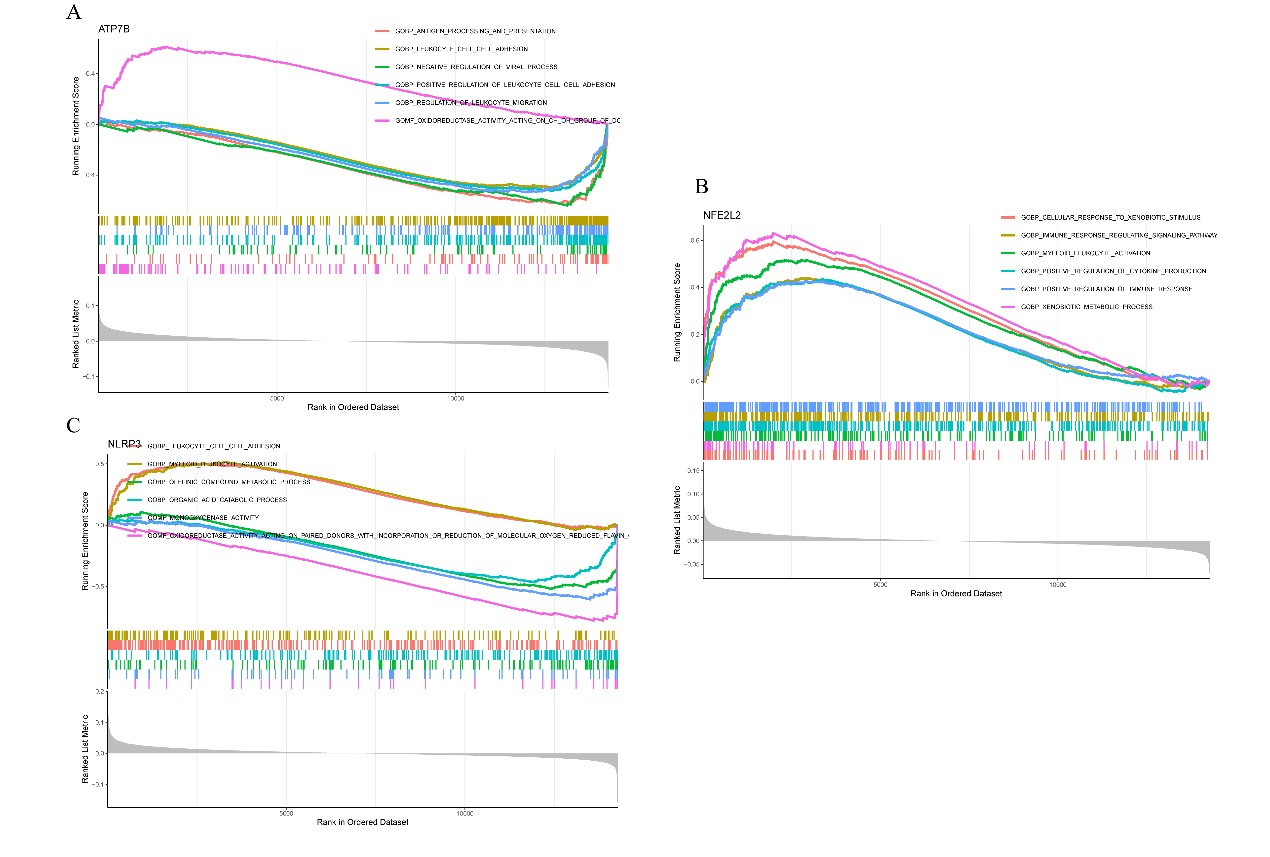
 GSEA result of GO enrichment for ATP7B(A), NFE2L2(B), and NLRP3(C).
